# Disturbances of Dynamic Function in Patients With Bipolar Disorder I and Its Relationship With Executive-Function Deficit

**DOI:** 10.3389/fpsyt.2020.537981

**Published:** 2020-09-24

**Authors:** Yan Liang, Xiaoying Jiang, Wenjing Zhu, Yonghui Shen, Fengfeng Xue, Yi Li, Zhiyu Chen

**Affiliations:** ^1^Department of Psychiatry, Hangzhou Seventh People’s Hospital, Hangzhou, China; ^2^Mental Health Center, Zhejiang University, School of Medicine, Hangzhou, China

**Keywords:** bipolar disorder, executive function, dynamic local activity, dynamic functional connectivity, posterior cingulate cortex, medial prefrontal cortex

## Abstract

Abnormity in brain regional function and inter-regional cooperation have been linked with the dysfunction during cognitive and emotional processing in bipolar disorder (BD) patients. Recent evidences have suggested that brain function is not static but temporal dynamic. In present study, we aimed to characterize the temporal dynamics of regional function and inter-regional cooperation in BD and its relationship with executive dysfunction, an important deficit in BD. Resting-state functional MRI was performed in patients with bipolar I disorder (BDI) (*n* = 18) and healthy controls (HCs, *n* = 19). We first assessed local-function temporal variety with dynamic amplitude of low-frequency fluctuation (dALFF). Region with significant inter-groups difference in dALFF was chosen as a seed to calculate inter-regions connective temporal variety with dynamic functional connectivity (dFC). The executive function was measured by Verbal Fluency Test (VFT). The relationship between executive function and brain dynamics were examined. Compared with HC, the BDI group showed decreased dALFF (less temporal variability) in the posterior cingulate cortex (PCC) and decreased dFC between PCC and medial prefrontal cortex (mPFC). The PCC-mPFC dFC was positively associated with VFT in BDI patients, but not in HC. These findings implicated the reduced temporal variability in local region and inter-regions cooperation in BDI, which may be a neural substrate of executive-function deficit in BDI.

## Introduction

Bipolar disorder (BD) is a common psychiatric disorder with remarkable mood instability, characterized by recurrent episodes of mania, hypomania, depression, and euthymic mood states (1). Bipolar I disorder and bipolar II disorder represent the most severe types of BD: the first former is characterized by alternating depressive and manic episodes of great severity that frequently requires hospitalization, while the latter is characterized by depressive and hypomanic episodes. Besides mood instability, neurocognitive dysfunction is another core feature of BD, which not only present in affective episodes, but also in euthymic periods. The neurocognitive dysfunctions involve multiple cognitive processes, including memory, attention, reward and executive function (2–5). Executive dysfunction is an important domain in BD, even considered as the specific deficit in BD. Importantly, executive dysfunction in BD are associated with poor social function (6, 7), delayed recovery (8) and higher readmission rate (9). However, the neural substrates of the executive dysfunction are still unclear for now.

Previous studies about the neural underpinnings of executive functions consistently implicated the key role of prefrontal cortex in healthy individuals (10). As a key node in emotion regulation network, prefrontal cortex certainly will contribute to the development of BD (11). Evidences from task-based fMRI studies have suggested abnormal activation in prefrontal cortex among BDI patients during executive tasks. However, these results are usually inconsistent. Strakowski SM et al. reported decreased activation in the lateral PFC in BDI during an executive task (12). In contrast, other studies indicated the hyperactivation of the lateral PFC among BD patients during verbal fluency task (VFT) (13, 14), a simple but class executive task. These inconsistent results may attribute to several factors, such as the type of BD and different tasks.

Recently, task-free fMRI (resting-state fMRI, RS-fMRI) provide a new insight to explore the neural substrate of executive dysfunction in BD. RS-fMRI could reveal the brain’s intrinsic neural activity in abnormal brain with blood oxygen level-dependent (BOLD) signals at rest. Zang Y et al. developed an index, amplitude of low-frequency fluctuation (ALFF), to detect the regional intensity of spontaneous activity (15). Studies with ALFF have found aberrant regional activity in several regions among patients with BD (16, 17). Notably, functional connectivity is another important index, which reflects inter-regional cooperation at rest. Mounting studies have identified abnormal functional connectivity and its relationship with emotional symptoms and executive deficits in BD (18, 19). Besides, the functional connectivity has been applied to reveal a host of intrinsic connectivity networks, referred to as brain networks, with distinct spatial and temporal profiles. The default mode network (DMN) is one of the most usually reported networks in psychiatric disorders, including BD. The DMN was initially identified as areas that consistently presented deactivation during most tasks and activation during resting state (20). Abnormal connectivity within DMN and inter-works connectivity of DMN with other networks have been frequently reported among patients with BD (21, 22).

Previous studies about functional connectivity mainly referred to the static functional connectivity (sFC), which is based on the assumption that the connection is spatial and temporal stationarity throughout the scanning period. This assumption may ignore the time-varying characteristics of connections, which have been proved by other measurements, such as electroencephalo-graph (EEG) (23, 24). To address this issue, recent studies with RS-fMRI developed dynamic analysis to estimate the variability of inter-region connections, i.e., dynamic functional connectivity (dFC). The credibility of the dFC has been demonstrated by evidence from simultaneous EEG/fMRI studies (25, 26).

Actually, several evidences have implicated the aberrant dFC among BD patients. Tanya T. Nguyen et al. reported the decreased dFC with a seed-seed wised approach (27). Although the findings are interesting, the seed-seed wised approach has several limitations, especially the seed-defined process. Recently, studies increasingly reported the dynamics of local brain activity among normal and dysfunctional brain at voxel level. Indeed, the local brain activity is supposed to be a reflection of mental activity (28), which may cause high time-varying (29). Multiple studies have found the abnormal temporal dynamics of local brain activity in affective disorder. For example, Li J. et al. computed the dynamic amplitude of low-frequency fluctuation (dALFF) to characterize the dynamics of local brain activity in depressed patients with suicidal ideation and found decreased brain dynamics in several limbic areas (30).

In the present study, we aimed at exploring the dynamic local activity and functional connectivity and its relationship with executive dysfunction in patients with BDI at voxel level. Based on the fact that the DMN plays a key role in the development of mood instability and executive dysfunction, we assumed that BDI patients present abnormal temporal variability of local region and inter-regions cooperations among key nodes in DMN, such as PCC and mPFC, as previous findings suggested. These decreased temporal variabilities may be associated with executive function deficit in BDI patients.

## Materials and Methods

### Participants

A total of 19 inpatients were recruited from the Hangzhou Seventh People’s Hospital, Hangzhou, China and diagnosed with bipolar disorder I (BDI) according to the Diagnostic and Statistical Manual of Mental Disorders, Fourth Edition (DSM-IV). The diagnosis was conducted by two psychiatrists based on structured clinical interviews. Patients would not be enrolled if the diagnosis is inconsistent between two psychiatrists. Patients would also be excluded due to the following exclusion criteria: (1) electroconvulsive therapy history during the last 6 months; (2) age over 65 years; (3) diagnosed with substance misuse, schizoaffective disorder or schizophrenia; (4) past or current neurological illness; (5) other contraindications of MRI scan administration; (6) too large head motion (>3 mm in translation or 3 in rotation). One patient was excluded because of large head motion, so 18 patients were included in the final analysis.

We also recruited 19 gender-, age-, and education-matched healthy subjects as controls *via* local advertisements. They were carefully screened through a diagnostic interview, the Structured Clinical Interview for DSM-IV (nonpatient edition), to rule out the presence of current or past psychiatric illness. Further exclusion criteria for the controls were any history of psychiatric illness in first-degree relatives and any current or past significant medical illness or mental disorders. This study was approved by the Hangzhou Seventh People’s Hospital Ethics Committee, and written informed consent was obtained from all participants.

### Clinical Evaluation

The Bech-Rafaelsen Mania Rating Scale (BRMS) was used to evaluate clinical symptoms for all participants during the 7-day period prior to the scan. The executive function was assessed by the verbal fluency test (VFT). During the VFT, participants were required to say as many words as possible describing an animal and a vegetable within 1 min, respectively. One point was scored when participants gave a correct term for either the correct description of an animal or a vegetable.

### MRI Data Acquisition

Resting-state and structural images of all participants were acquired at the Hangzhou Seventh People’s Hospital. Patients were instructed to keep their eyes closed and do not move and think as little as possible during the MRI scanning. Resting-state MRI scans were conducted under a 1.5 T MRI scanner (Signa HDxt 1.5 T, GE Healthcare, Buckinghamshire, UK) composed of 180 echo-planar imaging volumes with the following parameters: TR = 2000 ms; TE = 40 ms; flip angle = 85°; matrix size = 64 × 64, field of view = 240 × 240 mm; slice thickness = 3 mm; 28 continuous slices. Total acquisition of resting-state MRI lasted 6 min. A T1-weighted anatomical image was also acquired for each patient to further elucidate and discard gross radiological alterations. (TR = 9.5 ms; TE = 3.1 ms; flip angle = 20°; field of view = 240 mm × 240 mm; slice thickness = 1.2 mm).

### Functional Data Preprocessing

Functional MRI data were preprocessed with the Data Processing Assistant for Resting-State Functional MR Imaging toolkit (31). For each participant, we applied the following processing steps: discarding of the first 5 volumes to achieve a steady-state, slice timing correction, realignment, spatial normalization based on the unified segmentation of structural images, nuisance regressors with 24 Friston motion parameters, white matter high signal, cerebrospinal fluid signal and global signals as regressors, filter with a temporal band-pass of 0.01–0.1 Hz and spatial smoothing (Gaussian kernel = 4 mm × 4 mm × 4 mm). Finally, motion scrubbing was conducted using the method of cubic spline to minimize the influence of the time points with high motion [frame-wise displacement (FD) > 0.5], as well as one time point prior to, and two time points following each of these high motion time points. Finally, there are 9 frames with high motion for BDI group and 16 frames for healthy control (HC) group. The range of FD is between 0 and 4.62 for BDI group and between 0 and 2.22 for HC group.

dALFF and dFC were calculated using DynamicBC software with the sliding window approach (32). According to previous study, we chose 30 TRs (60 s) as the window length for resting-state dynamic analysis (30, 33), and the window was shifted by one TRs. In our data, the full-length time series was comprised of 175 TRs, so there were 145 windows for each participant. For the time series in each window, the ALFF map was obtained. The ALFF of each voxel was divided by the global mean ALFF value to normalize the global effects. To study the temporal variability of ALFF, we computed the variance of dALFF maps (standard deviation of ALFF at each voxel) across sliding-window dynamics.

The functionally abnormal region in local-activity dynamics (PCC, see Results section) was selected as seed to calculate dFC. The Pearson’s correlation coefficient of the PCC with all other voxels in brain was calculated and Fisher’s z-transformed, yielding a sliding-window z-value map. Then, for each participant, a set of sliding-window z-value maps were used to calculate the dFC map (standard deviation in z values at each voxel). Finally, the dFC maps were converted to z-values using Fisher’s r-to-z transformation to improve normality.

In our functional data preprocessing, we conducted global signal regression, which may increase tissue sensitivity and decrease motion dependency. However, the impact of global signal regression (GSR) on dynamic FC is obscure for now (34).

To probe the effect of GSR on dynamic FC, we add complementary analysis without regressing global regression during preprocessing.

### Statistical Analysis

The Pearson’s χ^2^ test was applied to compare sex differences and two-sample *t*-tests were applied to check for differences in age, educational years, clinical symptoms and executive-function performance between two groups. Voxel-wise two-sample *t*-tests with gender, age, educational years, and head motion indexed by FD as covariates were used to quantitatively compare the differences in the dALFF and dFC of posterior cingulate cortex (PCC, see below) the between two groups within the grey matter mask. All statistical maps were corrected using the Gaussian Random Field method at the threshold for voxel P < 0.001 and cluster *P* < 0.05. Then, we extracted ROI data to conduct correlation analysis. Pearson’s correlation analyses were performed to examine the associations between the brain dynamics and behavioral performance (clinical symptoms and executive-function performance). Significance was determined by *P* < 0.05 (two-tailed), with no correction.

## Results

### Demographic and Clinical Characteristic

Present study included 18 patients with BDI and 19 HCs. Demographic characteristic of the two groups are shown in **Table 1**. There was no significant difference between two groups in terms of age, gender, or head motion. Compared with the HCs, participants with BDI presented greater BRMS and lower VFT scores.

**Table 1:** Demographic and clinical characteristic.

	BDI	HC	^b^T value/χ^2^	p value
Gender (Male/Female)^a^	10/8	12/7	0.22	0.64
Age	31.67 ± 11.20	32.16 ± 10.35	0.14	0.89
Educational years	15.22 ± 2.07	15.68 ± 3.68	0.47	0.64
Age of onset	21.83 ± 8.61			
Number of hospitalization	6.67 ± 7.43			
Medication(patients number)				
Anticonvulsant	28			
Antipsychotics	22			
BRMS	23.89 ± 7.60			
VFT	18.17 ± 4.1	23.11 ± 5.44	3.10	0.004
FD value	0.11 ± 0.056	0.099 ± 0.009	0.88	0.385

a Data are presented as Mean ± Standard Deviation except Gender. ^b^Comparisons were performed with chi-square test for the variable of Gender and independent samples t-tests for other variables. BDI, bipolar disorder I; HC, healthy control; BRMS, Bech-Rafaelsen Mania Rating Scale; VFT, Verbal Fluency Test; FD, frame-wise displacement, used to evaluate head motion during scanning.

### Group Comparison of dALFF

**Figure 1:**
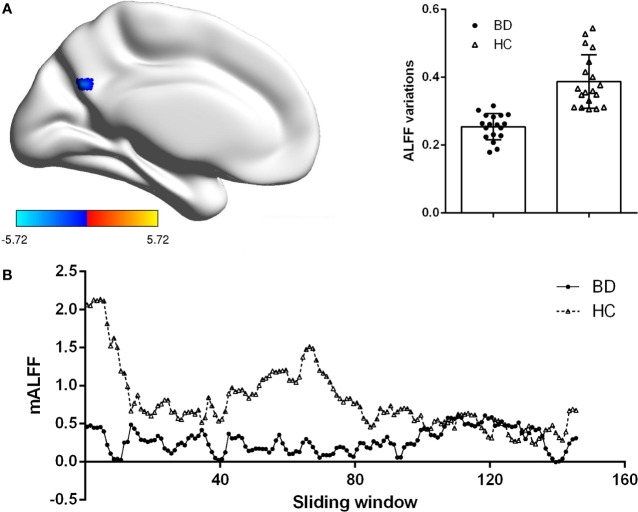
Group difference on the ALFF variations. **(A)** Patients BDI showed decreased ALFF variations (dALFF) in posterior cingulate cortex (PCC) than HC. Statistical map were corrected with Gaussian Random Field (GRF) method at threshold of voxel P < 0.001, cluster P < 0.05. Scatter plots present ALFF variations of each participant in two groups. **(B)** The mean ALFF were displayed for a single participant in the BDI group and a single participant in the HC group across all sliding windows.

We explored the difference of dALFF between the two groups based on voxel level. Compared with HCs, patients with BDI presented decreased dALFF in posterior cingulate gyrus (PCC) (voxel size = 22; peak coordinate = −3, −66, 30) (shown in **Figure 1**). No other regions with significant different dALFF were found between two groups.

### Group-Level Comparison of dFC Based on the Seed of PCC

**Figure 2:**
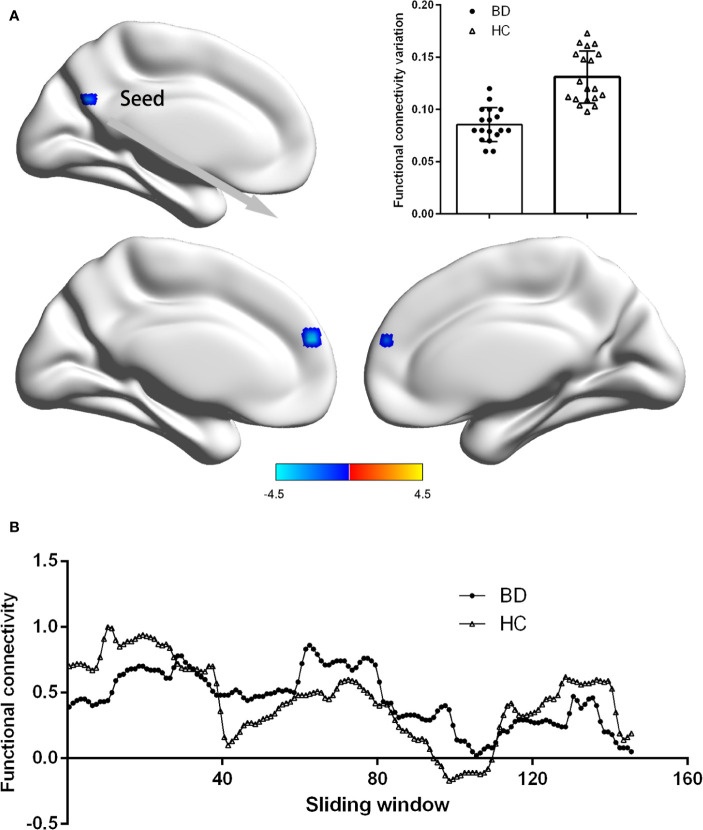
Group difference on the functional connectivity variations of PCC. **(A)** Patients BDI showed decreased functional connectivity variations (dFC) between PCC and medial prefrontal cortex (mPFC) than HC. Statistical map were corrected with Gaussian Random Field (GRF) method at threshold of voxel P < 0.001, cluster P < 0.05. Scatter plots present PCC-mPFC dFC of each participant in two groups. **(B)** The PCC-mPFC functional connectivity were displayed for a single participant in the BDI group and a single participant in the HC group across all sliding windows.

**Figure 2** showed the difference of dFC based on PCC between patients with BDI and HCs. Compared with HCs, depressive individuals presented decreased dFC between PCC and medial prefrontal cortex (mPFC) (voxel size = 35; peak coordinate = −3, −57, 25). No other regions with significant different dFC were found between two groups.

### Group-Level Comparison of dFC Based on the Seed of PCC

To probe the effect of GSR on dynamic FC, we add complementary analysis without regressing global regression during preprocessing. Our results suggested that after without GSR, the difference of dALFF and dFC were significant at the threshold for voxel P < 0.005, but not significant for voxel P < 0.001 (see **Supporting Informationl**, **Figure S1**).

### The Relationship Between dALFF and dFC and Behavioral Performance

**Figure 3:**
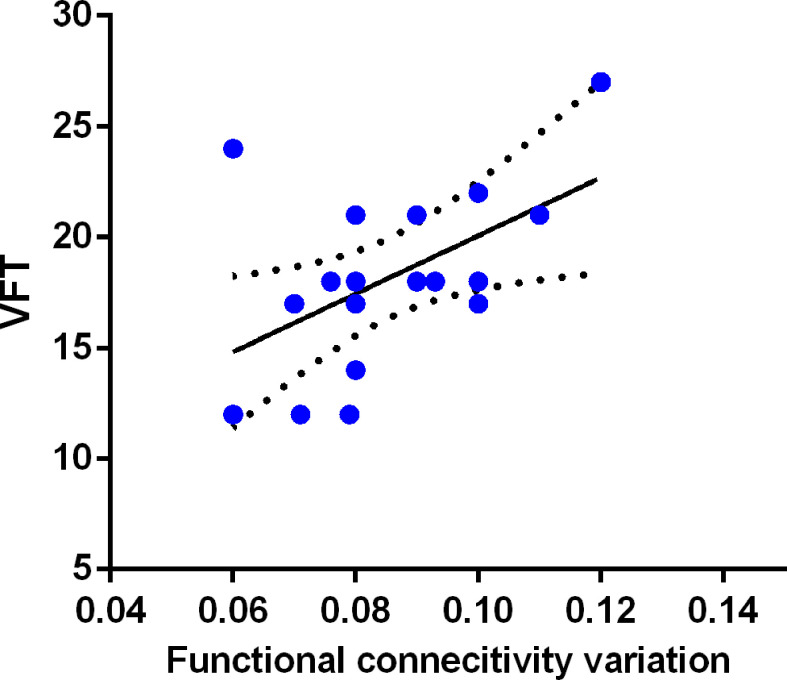
Relationship between PCC-mPFC dFC and executive function in BDI patients. The PCC-mPFC dFC was positively correlated with executive function evaluated by the Verbal Fluency Test (VFT) in patients with BDI.

A positive relationship (r = 0.521, p = 0.026) existed between right PCC-mPFC dFC and VFT scores among patients with BDI as shown in **Figure 3**. No significant correlation was found between PCC dALFF and VFT scores (r = −0.057, p = 0.822). There was also no significant correlation between clinical-symptoms severity and PCC dALFF or PCC-mPFC dFC (r = 0.052, p = 0.839; r = 0.055, p = 0.830).

## Discussion

In present study, we mainly aimed at exploring the temporal dynamics of resting-state local activity and functional connectivity in patients with BDI, as well as its relationship with clinical symptoms and executive function. Our results suggested that patients with BDI showed decreased dALFF (less temporal variability) in the PCC and decreased dFC between PCC and mPFC. The PCC-mPFC dFC was positively associated with VFT in BDI patients, but not in HC. These findings suggest the reduced temporal variability in local region and inter-regions cooperations in BDI, which may be a neural substrate of executive-function deficit in BDI.

As a common and less time-consuming cognitive measurement, the VFT has been applied to investigate executive function. Present study found that BDI patients displayed weaker scores in the VFT, i.e., executive deficits. Prior literature has reported euthymic BDI were impaired in verbal memory and executive functioning which can be considered as a potential endophenotype of BDI (35–37). However, it must be admitted that the VFT may reflect reasoning ability and perceptual speed, only a fraction of executive function, which encompass various aspects, such as response inhibition, working memory and cognitive flexibility (38). Research with more multiple tests is required to replicate present findings.

The PCC is a key node in the DMN and was implicated in self-referential and reﬂective processes, such as episodic memory retrieval, images, and emotions (39). Although results were inconsistent, previous studies have linked DMN abnormalities with multiple mood disorders, such as depression and BDI (40–42). Indexed with static ALFF, the DMN abnormalities have been reported by plentiful studies. For example, Zhong et al. has reported that BD patients showed decreased ALFF in precuneus and PCC which are nodes of DMN (43). These abnormalities may underlie cognitive and affective processing problems in psychiatric conditions (41, 44). Consistent with previous studies, our results suggested the abnormal DMN temporal dynamics of spontaneous activity in BDI individuals. Unfortunately, we did not find the relationship between PCC local-activity dynamics (dALFF) and clinical symptoms or executive dysfunctions. Small sample size, high sample heterogeneity may be the potential factors which contribute to this confused condition. More importantly, this embarrassed status may also related with the fact that BDI is as a disorder driven by different regions interconnected dysregulation, rather than a dysfunction in one single brain area (45, 46).

Indeed, our results revealed the decreased PCC-mPFC connection variations in patients with BDI. The mPFC has been reported to be involved in several executive processing, such as response inhibition, inhibitory control, and task switching (10, 47, 48). Compared with healthy individuals, patients with BDI have been showed hypoactivation in mPFC (49). Evidence from resting-state connectivity studies also suggested the decreased connection between mPFC and PCC in BDI patients (50). Individuals with reduced PCC-mPFC connectivity exhibited impairment in executive function (51). In addition, the mPFC is also component of DMN and the reduced connectivity within DMN in patients with BDI has been frequently reported (51–53). The dysfunctions of DMN have been closely linked with both emotional and executive impairments of BDI (22, 54).

Besides static connection, the connective dynamics may be also important for the executive function and emotional regulation. Kucyi et al. suggested dFC within DMN is positively associated with emotion control (55). Abnormal intra- and internetwork connectivity variability are linked with mood disorders (56). Inter-network connectivity dynamics also contribute to cognitive flexibility (57), which is a key element of executive function. Consistent with these findings, our results revealed that the decreased PCC-mPFC connectivity dynamics in BDI is positively associated with the executive performance. Indeed, the relationship between PCC-mPFC connectivity dynamics and executive impairments in BDI patients has been indicated by previous study based on region-of-interest (ROI) level (27). As borderless regions, it is difficult to define accurately PCC or mPFC that may limit the strength of evidence. To improve this situation, we first explore the abnormal dynamics of spontaneous activity at voxel level. Intriguingly, we found reduced PCC spontaneous activity variety and further revealed reduced PCC-mPFC connectivity, which is in keeping with the fact that the PCC is an important node in interacting with other brain networks and optimizing cognitive function (58).

Intriguingly, several studies reported increased dynamic FC in major depression (42), which is converse with our results in BDI. These findings may be a potential biomarker for differentiating the two diseases. Indeed, as two related but distinct diseases, major depression and BD patients may present disparate brain-function temporal dynamics. However, there is no uniform conclusion about the common or specific alterations of brain-function temporal dynamics (56, 59). Further studies included BD I and II type and major depression to explore the differentiating signatures of brain-function temporal dynamics.

Several limitations in present study are worthy of discussion. First, our study enrolled small-size sample. Larger samples in future are needed to confirm current findings. Second, all patients included in the present study were administrated with drugs; we still cannot eliminate the effects of drugs on our findings. Third, the scanning quality is not most suitable for dynamic analysis, such as long repetition time (2 s), insufficient time points (180) and inferior magnetic field intensity (1.5 T). Finally, present study only included BDI patients during mania episode but not the depressive episode, which impeded us to exploring that the reduced PCC-mPFC connectivity variety is a state or trait biomarker of BDI.

## Data Availability Statement

The raw data supporting the conclusions of this article will be made available by the authors, without undue reservation, to any qualified researcher.

## Ethics Statement

The studies involving human participants were reviewed and approved by Hangzhou Seventh People’s Hospital Ethics Committee. The patients/participants provided their written informed consent to participate in this study.

## Author Contributions

ZC supervised present study. YaL and XJ performed the analysis and wrote paper. WZ, YS, FX, and YiL helped to collect data.

## Funding

This work was supported by funding from the Key Medical and Health Science and Technology Projects of Hangzhou (2015Z07) and Social development fund of science and technology bureau of Hangzhou (20191203B120).

## Conflict of Interest

The authors declare that the research was conducted in the absence of any commercial or financial relationships that could be construed as a potential conflict of interest.
