# Supplementary figures and images for: Disturbances of Dynamic Function in Patients With Bipolar Disorder I and Its Relationship With Executive-Function Deficit
Source: Front Psychiatry. 2020 Sep 24;11:537981. doi: 10.3389/fpsyt.2020.537981 (PMC7542231; doi:10.3389/fpsyt.2020.537981)

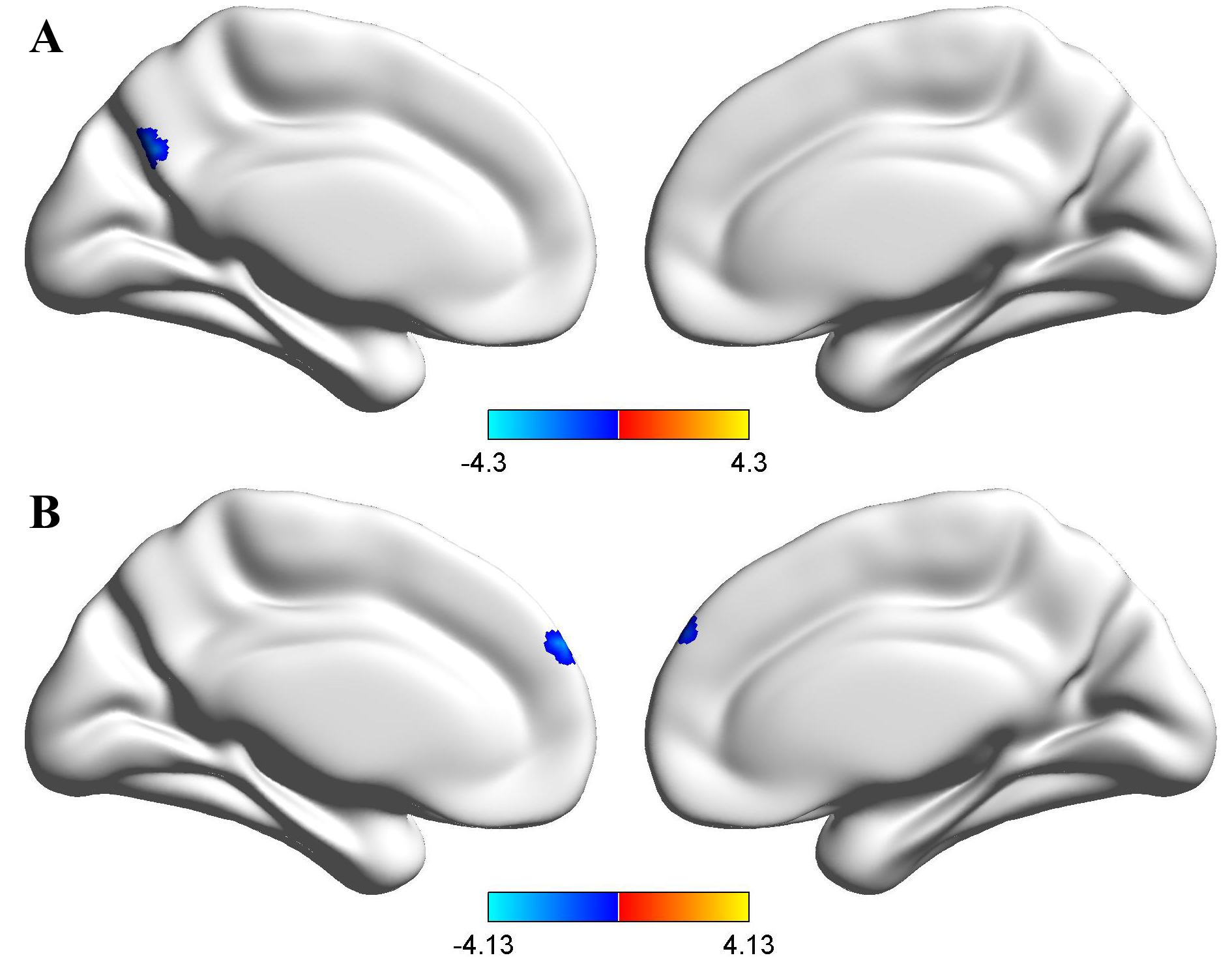

Supplement: Supplementary file 1 [file Image_1.tif]
